# Epigenetic profiling reveals a subset of pediatric-type glioneuronal tumors characterized by oncogenic gene fusions involving several targetable kinases

**DOI:** 10.1007/s00401-022-02492-7

**Published:** 2022-09-07

**Authors:** Philipp Sievers, Martin Sill, Daniel Schrimpf, Dennis Friedel, Dominik Sturm, Maria Gardberg, Kathreena M. Kurian, Lenka Krskova, Ales Vicha, Tina Schaller, Christian Hagel, Zied Abdullaev, Kenneth Aldape, Thomas S. Jacques, Andrey Korshunov, Wolfgang Wick, Stefan M. Pfister, Andreas von Deimling, David T. W. Jones, Felix Sahm

**Affiliations:** 1 Department of Neuropathology, Institute of Pathology, University Hospital Heidelberg, Heidelberg, Germany; 2 Clinical Cooperation Unit Neuropathology, German Consortium for Translational Cancer Research (DKTK), German Cancer Research Center (DKFZ), Heidelberg, Germany; 3 Hopp Children’s Cancer Center Heidelberg (KiTZ), Heidelberg, Germany; 4 Division of Pediatric Neurooncology, German Cancer Consortium (DKTK), German Cancer Research Center (DKFZ), Heidelberg, Germany; 5 Department of Pediatric Oncology, Hematology, Immunology and Pulmonology, University Hospital Heidelberg, Heidelberg, Germany; 6 Division of Pediatric Glioma Research, German Cancer Research Center (DKFZ), Heidelberg, Germany; 7 Department of Pathology, Turku University Hospital, Turku, Finland; 8 Institute of Biomedicine, University of Turku, Turku, Finland; 9 Brain Tumour Research Centre, Bristol Medical School, University of Bristol, Bristol, UK; 10 Prague Brain Tumor Research Group, Second Faculty of Medicine, Charles University and University Hospital Motol, Prague, Czech Republic; 11 Department of Pathology and Molecular Medicine, Second Faculty of Medicine, Charles University and University Hospital Motol, Prague, Czech Republic; 12 Department of Pediatric Haematology and Oncology, Second Faculty of Medicine, Charles University and University Hospital Motol, Prague, Czech Republic; 13 Pathology, Medical Faculty, University of Augsburg, Augsburg, Germany; 14 Institute of Neuropathology, University Medical Center Hamburg-Eppendorf, Hamburg, Germany; 15 Laboratory of Pathology, Center for Cancer Research, National Cancer Institute, National Institutes of Health, Bethesda, MD USA; 16 Developmental Biology and Cancer Research and Teaching Department, UCL Great Ormond Street Institute of Child Health, London, UK; 17 Department of Histopathology, Great Ormond Street Hospital for Children, NHS Foundation Trust, London, UK; 18 Clinical Cooperation Unit Neurooncology, German Consortium for Translational Cancer Research (DKTK), German Cancer Research Center (DKFZ), Heidelberg, Germany; 19 Department of Neurology and Neurooncology Program, National Center for Tumor Diseases, Heidelberg University Hospital, Heidelberg, Germany

Glioneuronal tumors (GNTs) are a diverse group of central nervous system (CNS) neoplasms that primarily affects children and young adults [6]. Their histopathological diagnosis can be extremely challenging due to overlapping morphological features among the different (sub-)types. In recent years, the use of next-generation sequencing and DNA methylation arrays revealed a large spectrum of different types of GNTs that are often characterized by a unique (epi-)genetic profile [2–5, 12, 13]. However, the molecular landscape of GNT is far from being exhaustively described. Interestingly, the vast majority of GNTs are driven by one of a variety of aberrations in the mitogen-activated protein kinase (MAPK) signaling pathway, including mutations, fusions or structural rearrangements in *BRAF*, *NF1*, *FGFR1* or *NTRK1/2/3*, and other rarer alterations [1, 3, 8, 11, 12]. Aberrant activation of the MAPK pathway is not only important from a diagnostic perspective, it also offers therapeutic opportunities since inhibitors are frequently available [9].

To identify novel epigenetic subgroups of GNTs, we used an unsupervised visualization approach with a comprehensive dataset of DNA methylation profiles covering the entire spectrum of existing molecular CNS tumor classes [2]. These analyses revealed a specific cluster of tumors (*n* = 14) with varying histological features of different GNT types (Fig. 1a). Clinicopathological characteristics are summarized in Fig. 1b and supplementary table 1 (online resource). Analysis of copy-number variations derived from DNA methylation array data indicated structural aberrations affecting the gene locus of different targetable kinases (Fig. 1b, c). Subsequent transcriptome and DNA sequencing [10, 14] in 12/14 of the cases confirmed oncogenic gene fusions involving several kinases including the *NTRK1/2/3*, *FGFR1/3*, *MET*, *RET* and *RAF1* genes. Of note, seven of the cases harbored rearrangements involving the *NTRK* gene family. For the most common partner (*n* = 5), *NTRK2* was fused downstream of either *AGAP1* (*n* = 2), *KCTD16* (*n* = 1), *SPECC1L* (*n* = 1) or *KIF5B* (*n* = 1). Single cases showed an *ARHGEF11::NTRK1* fusion or *ETV6::NTRK3* fusion. Genetic alterations within the FGFR signaling pathway were seen in two of the cases, with one case showing an *FGFR1::TACC1* fusion and another an *FGFR3::TACC3* fusion, both rearrangements reported in particular in extraventricular neurocytoma [7, 12]. In addition, oncogenic gene fusions of *ZMIZ1::RET*, *GOLGA4::MET* and *QKI::RAF1* were observed. Apart from a homozygous deletion of *CDKN2A/B* observed in one of the cases (Supplementary Table 1, online resource), no other relevant aberration was detected. These data suggest a remarkably wide range of different gene fusions that drive tumors within this epigenetic group and in parallel highlights attractive therapeutic targets in particular for patients with incomplete surgical resection or tumor progressions.

**Fig. 1:**
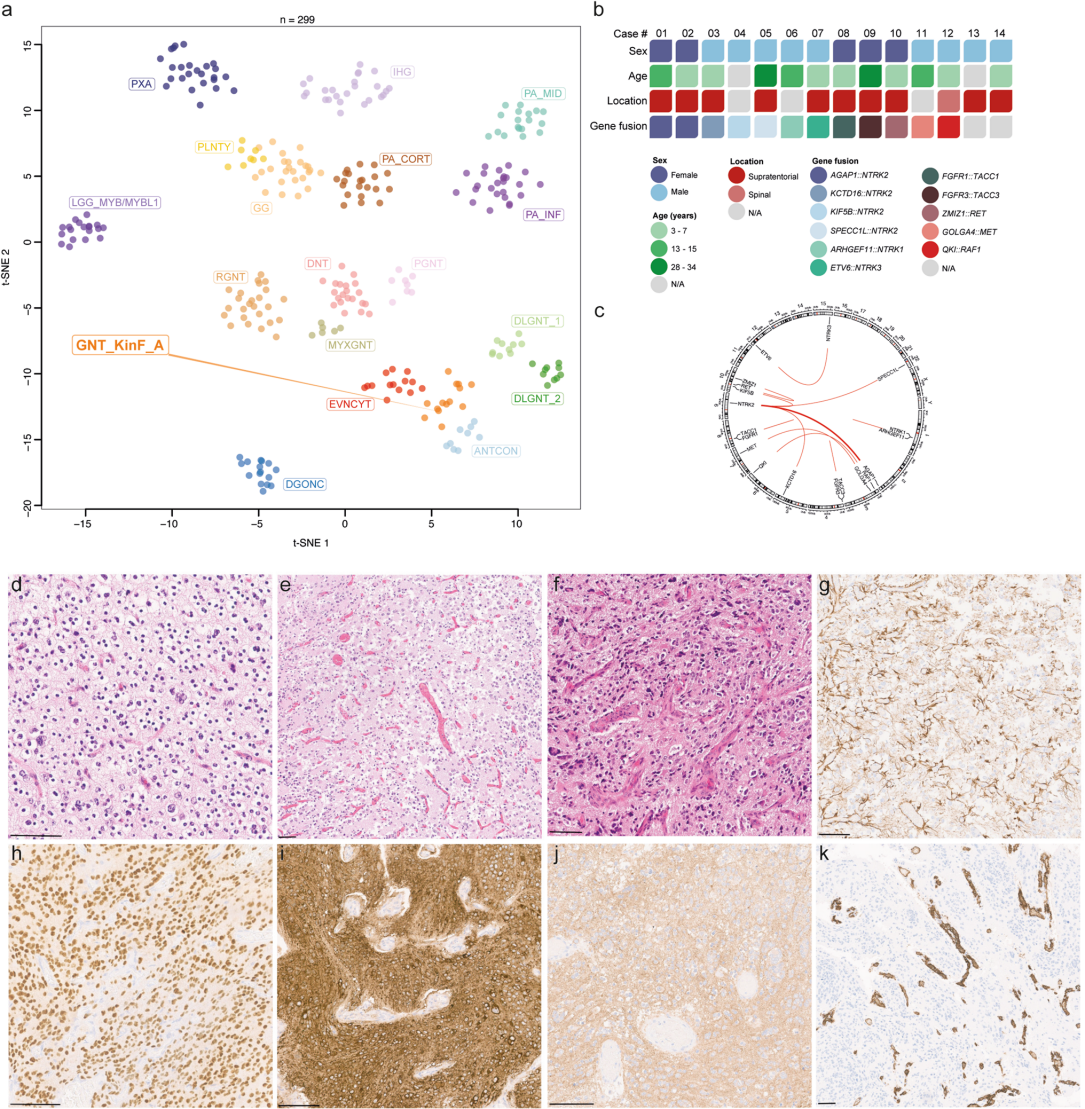
Unsupervised, nonlinear t-distributed stochastic neighbor embedding (t-SNE) projection of DNA methylation array profiles from 299 tumors. DNA methylation profiling reveals a molecular distinct group of glioneuronal tumors (GNT_KinF_A; **a**). Reference DNA methylation classes: dysembryoplastic neuroepithelial tumor (DNT), rosette-forming glioneuronal tumor (RGNT), diffuse leptomeningeal glioneuronal tumor subtype 1 (DLGNT_1), diffuse leptomeningeal glioneuronal tumor subtype 2 (DLGNT_2), extraventricular neurocytoma (EVNCYT), papillary glioneuronal tumor (PGNT), ganglioglioma (GG), polymorphous low-grade neuroepithelial tumor of the young (PLNTY), myxoid glioneuronal tumor, PDGFRA-mutant (MYXGNT), diffuse glioneuronal tumor with oligodendroglioma-like features and nuclear clusters (DGONC), anaplastic neuroepithelial tumor with condensed nuclei (ANTCON), angiocentric glioma MYB/MYBL1-altered (LGG_MYB/MYBL1), pilocytic astrocytoma hemsipheric (PA_CORT), pilocytic astrocytoma infratentorial (PA_INF), pilocytic astrocytoma midline (PA_MID), pleomorphic xanthoastrocytoma (PXA) and infant-type hemispheric glioma (IHG). Summary of clinical characteristics and key molecular findings in the 14 tumors investigated (**b**). Circos plot of the different gene fusions detected in the series (lines link fusion gene partners according to chromosomal location; **c**). Histologically, tumors show a moderate to high increase in cellular density of largely monomorphic (**d**–**e**) or slightly pleomorphic neoplastic cells (**f**). An oligodendroglial morphology with perinuclear halos is focally present in most of the tumors (**d**). Immunohistochemistry for GFAP is largely restricted to reactive astrocytes or a minor proportion of neoplastic cells (**g**). Tumor cells show immunoreactivity of OLIG2 (**h**), MAP2 (**i**), and synaptophysin (**j**). CD34 expression is restricted to the vessels (**k**). Scale bars 100 µm

The nine male and five female patients ranged in age at time of initial diagnosis from 3 to 34 years (*n* = 12; mean age 11.2 years). Tumors were located supratentorially (*n* = 10), with the exception of one case located in the spinal cord (Fig. 1b and Supplementary Table 1, online resource). Due to the diverse origins and the retrospective nature of the series, availability of clinical data (in particular in terms of patient outcome) was restricted for some of the cases and did not allow a reliable assessment of the malignancy of the tumors. Histologically (*n* = 10), the tumors shared a moderate to high increase in cellular density of largely monomorphic or slightly pleomorphic neoplastic cells (Fig. 1d–f). Only one of the tumors was characterized by a more pronounced cellular pleomorphism (Fig. 1f). The tumor cells typically had round to oval, partly hyperchromatic nuclei with prominent nucleoli (Fig. 1d–e). An oligodendroglial morphology with perinuclear halos was seen in the majority of the tumors (*n* = 7; Fig. 1d). In one case, spindle-shaped cells were observed focally. About half of the tumors (*n* = 6) focally showed perivascular rosettes, mostly together with small neuropil islands. Calcifications were seen in a small number of tumors (*n* = 2). Focal reactive vascular proliferation was detected in only two of the cases (Fig. 1f). Necrosis was not observed. Mitotic activity was absent or low, with the exception of two cases exhibiting a slightly higher rate of up to 0.8 and 1.7 mitosis per mm^2^. Immunoreactivity for GFAP was largely restricted to reactive astrocytes or a minor proportion of neoplastic cells (Fig. 1g). Tumor cells showed immunoreactivity of OLIG2, MAP2 and synaptophysin (Fig. 1h–j). Several tumors showed focal positivity for NeuN. CD34 expression was restricted to the vessels (Fig. 1k). The proliferation index (Ki-67) ranged from 1 to 20%. A summary of the morphological and immunohistochemical features of the tumors are given in Supplementary Table 2 (online resource).

Together, these findings suggest a molecularly distinct group of pediatric-type GNT characterized by oncogenic activation of different kinases. Although enriched for gene fusions involving the *NTRK* gene family, tumors within this epigenetic group show a remarkable spectrum of different rearrangements including very rare events in primary CNS tumors such as *RAF1* and *RET* fusions. Given their morphological overlap with other GNTs and the lack of a pathognomonic alteration, we provisionally suggest the term ‘glioneuronal tumor kinase-fused’ (GNT_KinF_A) to describe this novel group of tumors. In addition, our findings emphasize the potential benefit of molecular profiling to identify targetable alterations in GNTs.

## Supplementary Information

Below is the link to the electronic supplementary material.

Supplementary file1 (DOCX 1426 KB)

Supplementary file2 (XLSX 15 KB)
