# Supplementary material for: Epigenetic profiling reveals a subset of pediatric-type glioneuronal tumors characterized by oncogenic gene fusions involving several targetable kinases
Source: Acta Neuropathol. 2022 Sep 7;144(5):1049–52. doi: 10.1007/s00401-022-02492-7 (PMC9547789; doi:10.1007/s00401-022-02492-7)

**Supplementary materials and methods**

**Sample collection**

Patient tumor samples and retrospective clinical information were provided by multiple national and international collaborating centers and collected at the Department of Neuropathology of the University Hospital Heidelberg (UKHD, Germany) and German Cancer Research Center (DKFZ, Heidelberg, Germany). Sample selection was based on unsupervised visualization (t-distributed stochastic neighbor embedding (t-SNE) of genome-wide DNA methylation array data that revealed a molecularly distinct group of tumors forming a cluster separate from all established tumor types. Furthermore, DNA methylation array data of numerous well-characterized reference samples representing CNS tumors were used for comparative analyses. Detailed descriptions of the reference DNA methylation classes are outlined under (<https://www.molecularneuropathology.org>). Sample collection and processing and data collection were performed in accordance with local ethics regulations and approval.

**DNA methylation array processing and copy‑number profiling**

Genome-wide DNA methylation profiling of all samples was performed using the Infinium MethylationEPIC (EPIC) BeadChip (Illumina, San Diego, CA, USA) or Infinium HumanMethylation450 (450k) BeadChip array (Illumina) according to the manufacturer’s instructions and as previously described[1]. Raw data were generated at the Department of Neuropathology of the University Hospital Heidelberg, the Genomics and Proteomics Core Facility of the DKFZ or at respective international collaborator institutes, using both fresh-frozen and FFPE tissue samples. All samples were checked for duplicates by pairwise correlation of the genotyping probes on the 450k/EPIC array.

**Targeted next-generation sequencing analysis**

Capture-based next-generation DNA sequencing was performed on a NextSeq 500 or NovaSeq 6000 instrument (Illumina) as previously described[2] using a custom brain tumor panel covering the entire coding and selected intronic and promoter regions of 170 genes of particular relevance in central nervous system tumors. Sequence reads were mapped to the reference human genome build GRCh37 (hg19) using the Burrows-Wheeler aligner (BWA).

**RNA sequencing**

RNA was extracted from FFPE tissue samples. RNA sequencing for the purpose of gene fusion detection of samples was performed on a NextSeq 500 (Illumina) with 75 bp paired-end reads as previously described[3]. Fastq files from transcriptome sequencing were used for de novo annotation of fusion transcripts using the Arriba (v1.2.0) algorithm[4] with standard parameters.

I**mmunohistochemistry**

Immunohistochemical staining was performed on a Ventana BenchMark ULTRA Immunostainer using the ultraView Universal DAB Detection Kit (Ventana Medical Systems, Tucson, AZ, USA). Antibodies were directed against: glial fibrillary acid protein (GFAP; Z0334, rabbit polyclonal, 1:1000 dilution, Dako Agilent, Santa Clara, CA, USA), oligodendrocyte lineage transcription factor 2 (OLIG2; clone EPR2673, rabbit monoclonal, 1:50 dilution, Abcam, Cambridge, UK), MAP2 (clone HM-2, mouse monoclonal, 1:15000 dilution, Sigma-Aldrich, St. Louis, MO, USA), Synaptophysin (clone MRQ-40, rabbit monoclonal, 1:160 dilution, Cell Marque Corp., Rocklin, CA, USA), NeuN (clone A60, mouse monoclonal, 1:100 dilution, Millipore, Burlington, MA, USA), CD34 (clone QBEnd/10, mouse monoclonal, Ventana Medical Systems), and Ki-67 (clone MIB-1, mouse monoclonal, 1:100 dilution, Dako Agilent).

**References to supplementary methods**

1. Capper D, Jones DTW, Sill M, Hovestadt V, Schrimpf D, Sturm D, et al. DNA methylation-based classification of central nervous system tumours. Nature. 2018;555(7697):469-74. doi: 10.1038/nature26000.

2. Sahm F, Schrimpf D, Jones DT, Meyer J, Kratz A, Reuss D, et al. Next-generation sequencing in routine brain tumor diagnostics enables an integrated diagnosis and identifies actionable targets. Acta Neuropathol. 2016;131(6):903-10. doi: 10.1007/s00401-015-1519-8.

3. Stichel D, Schrimpf D, Casalini B, Meyer J, Wefers AK, Sievers P, et al. Routine RNA sequencing of formalin-fixed paraffin-embedded specimens in neuropathology diagnostics identifies diagnostically and therapeutically relevant gene fusions. Acta Neuropathol. 2019;138(5):827-35. doi: 10.1007/s00401-019-02039-3.

4. Uhrig S, Ellermann J, Walther T, Burkhardt P, Frohlich M, Hutter B, et al. Accurate and efficient detection of gene fusions from RNA sequencing data. Genome Res. 2021;31(3):448-60. doi: 10.1101/gr.257246.119.

**Supplementary Fig. 1** Visualization of the different fusion genes detected by RNA sequencing.


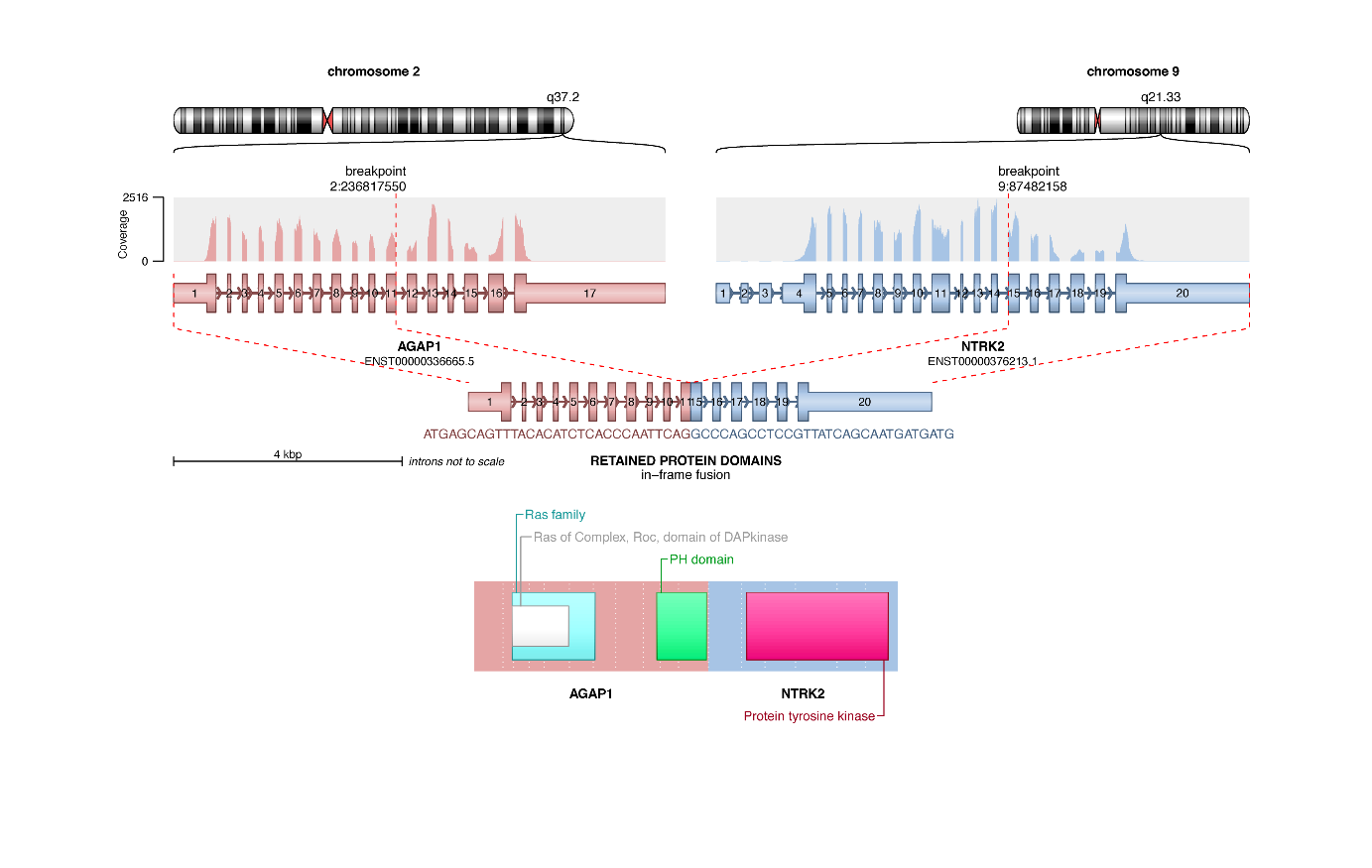


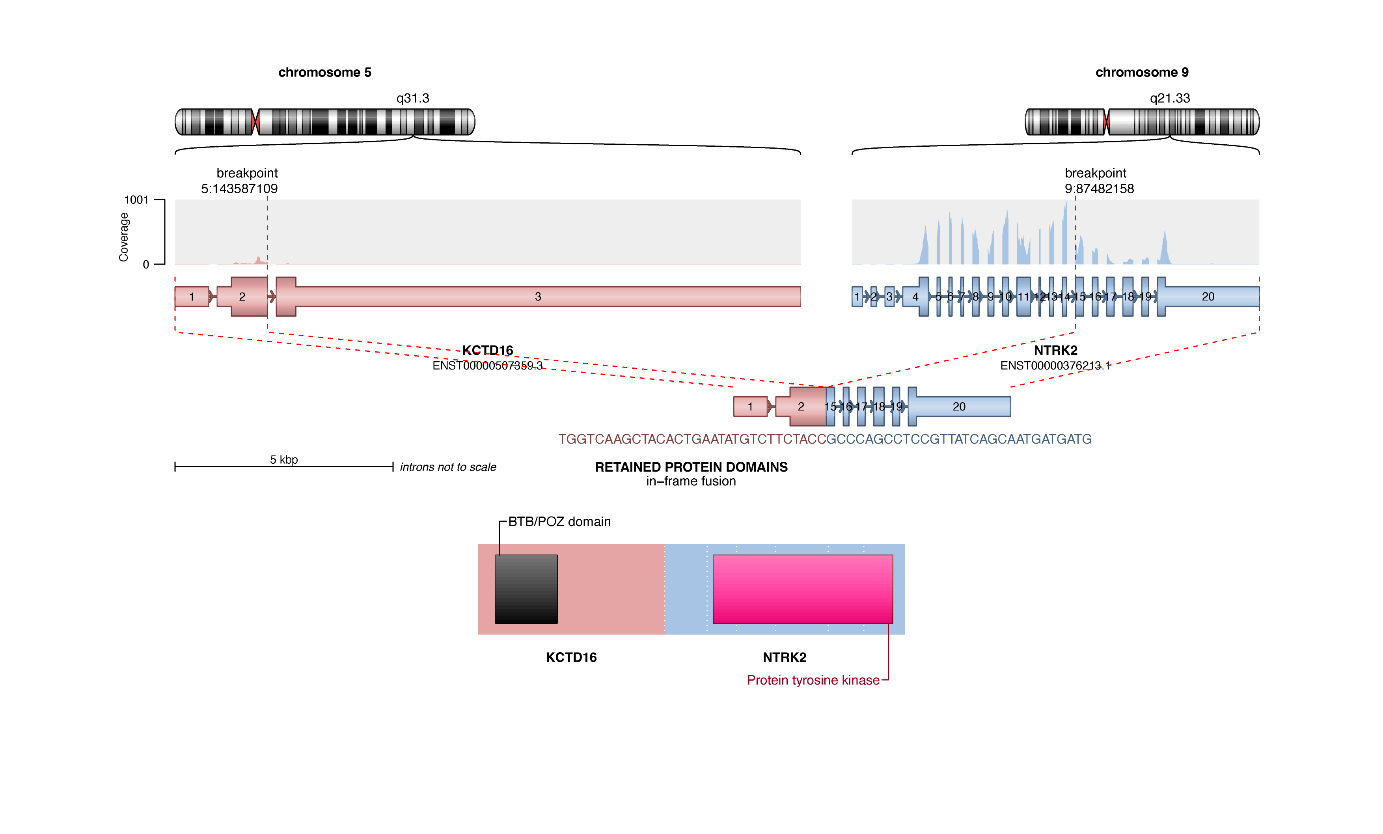


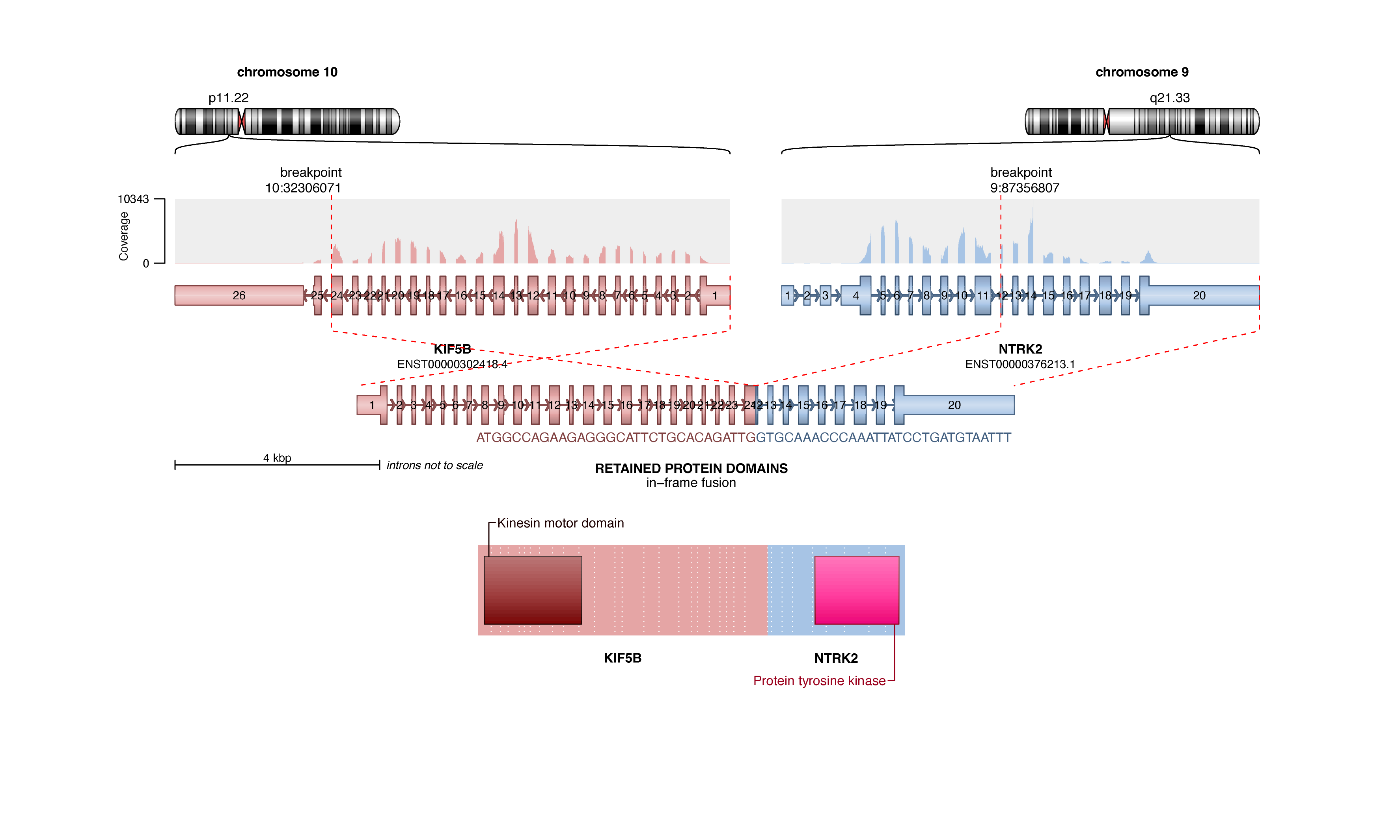


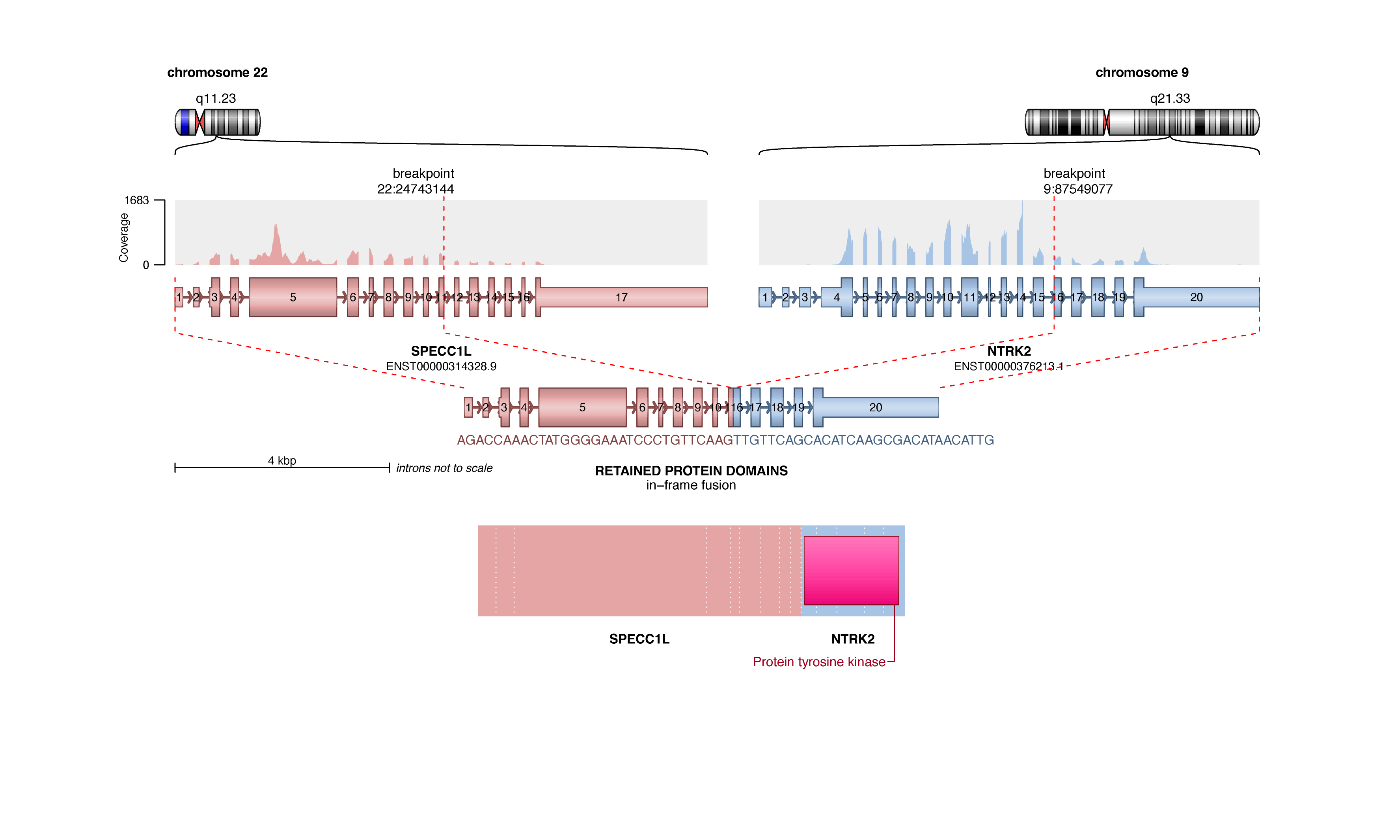


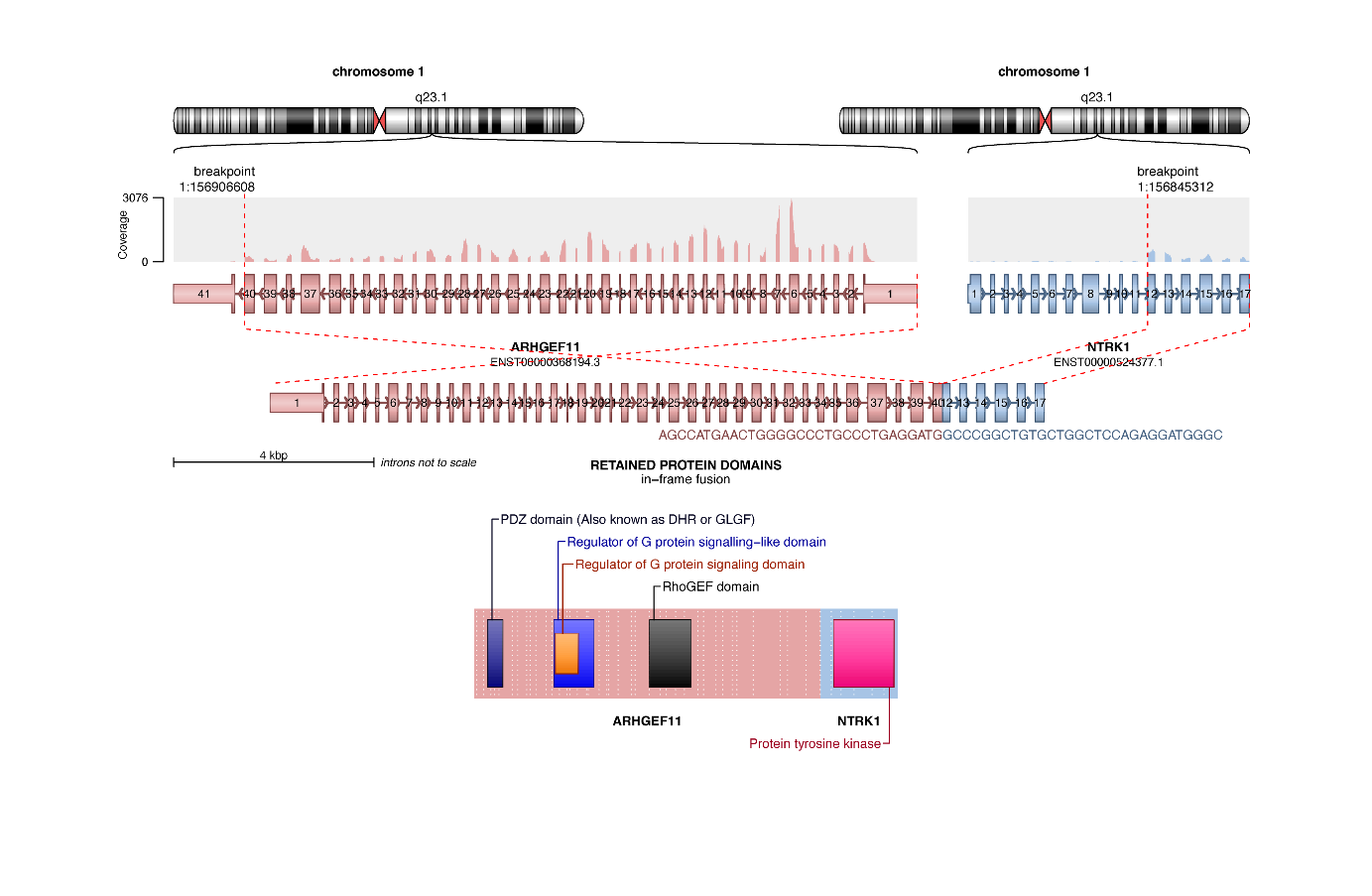


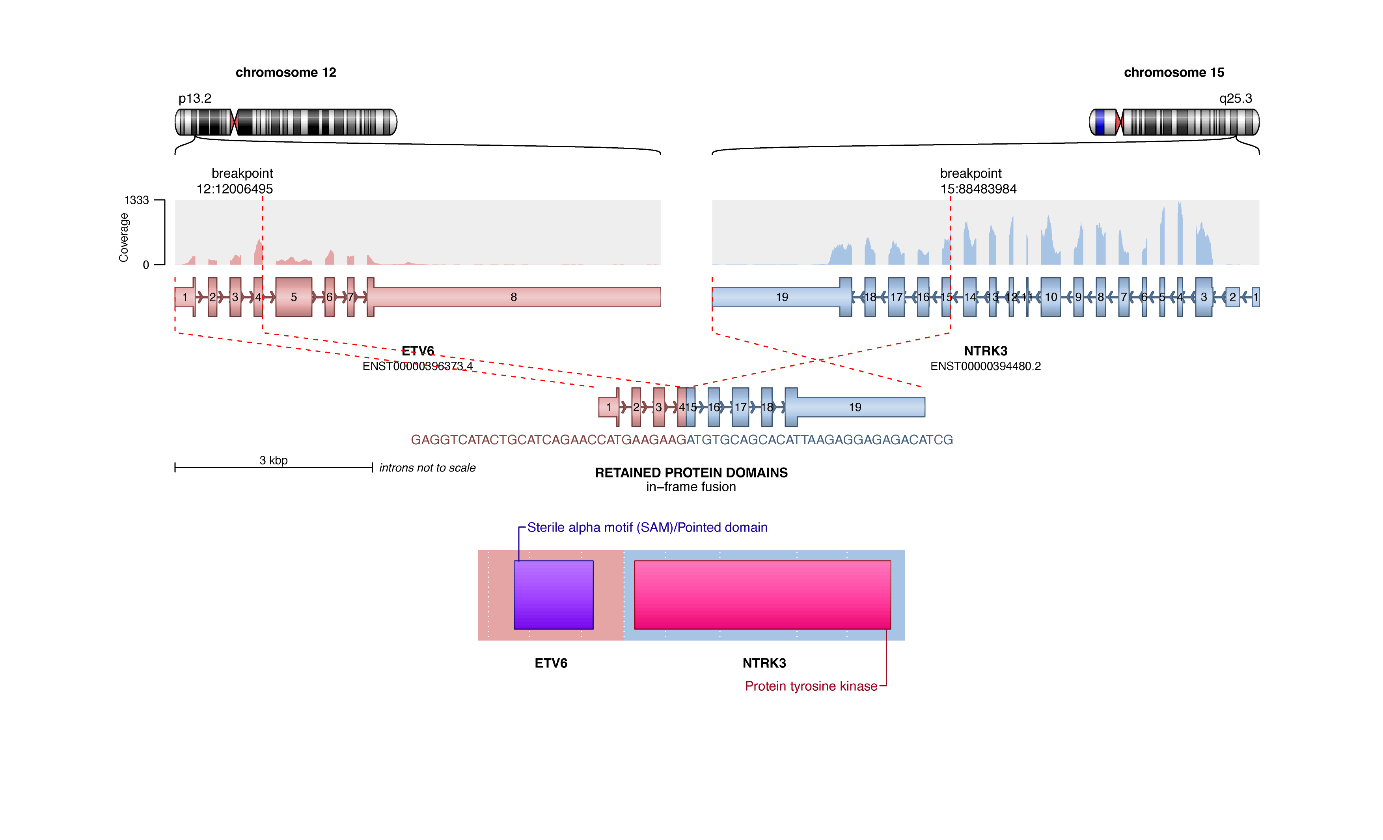


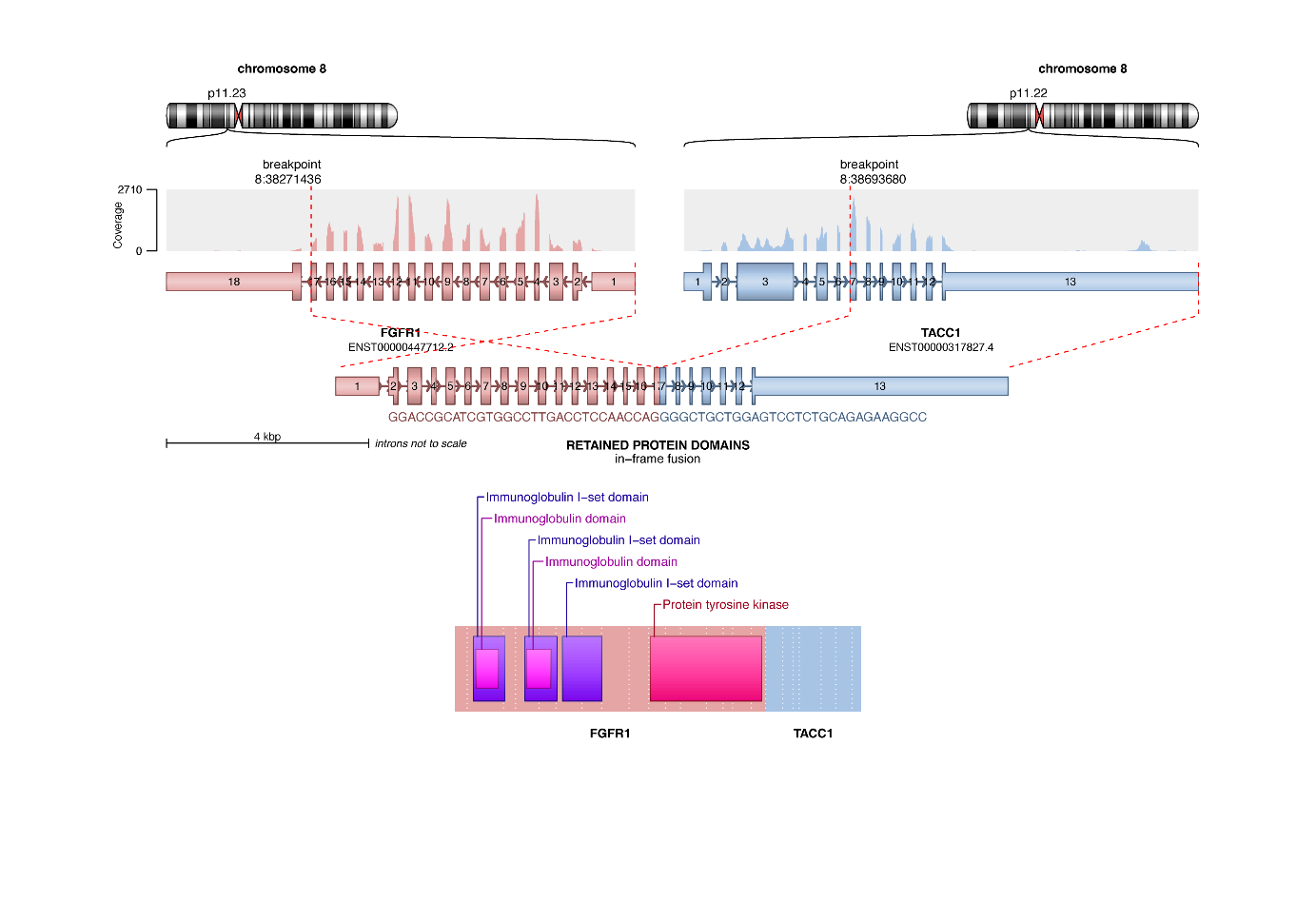


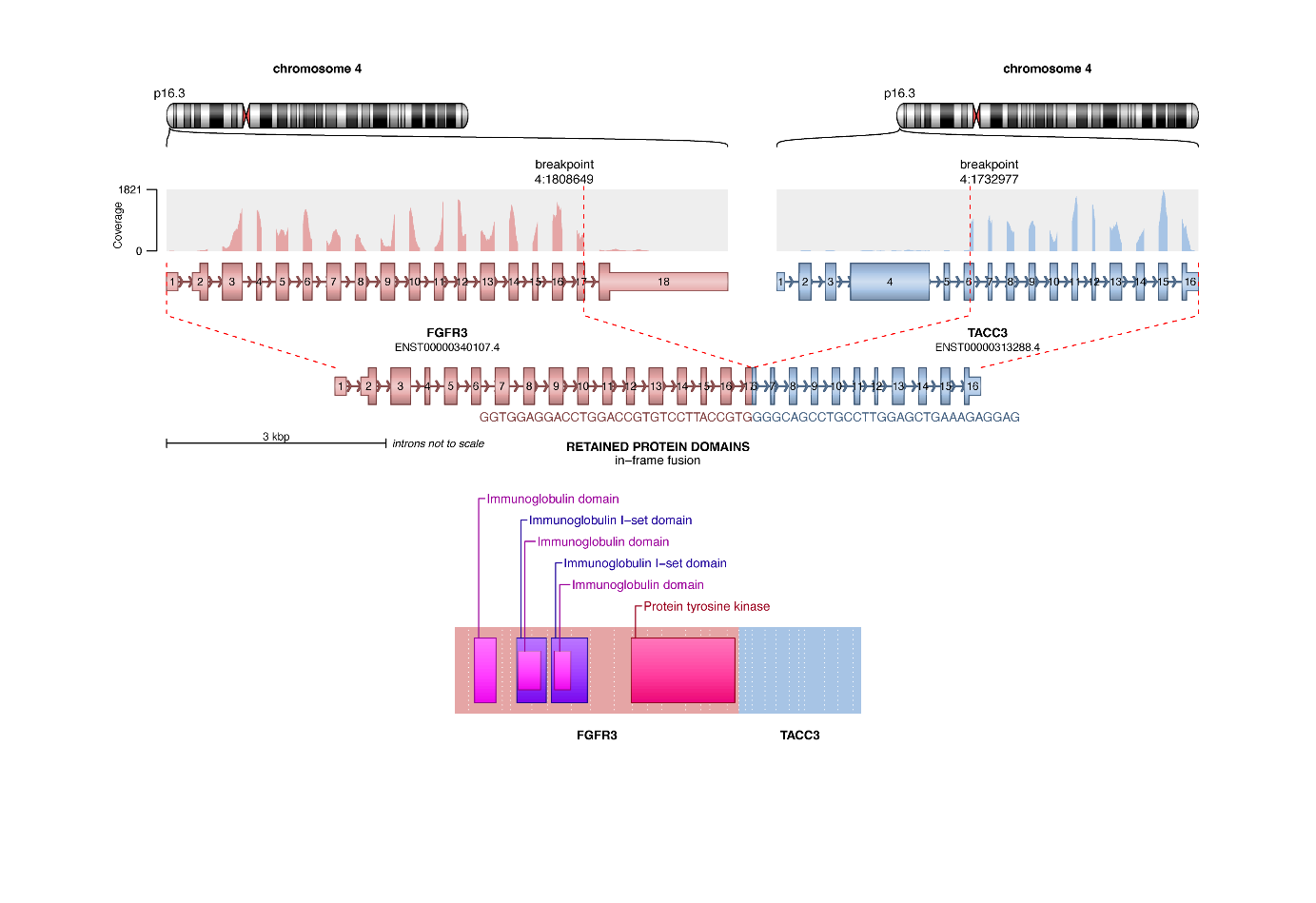


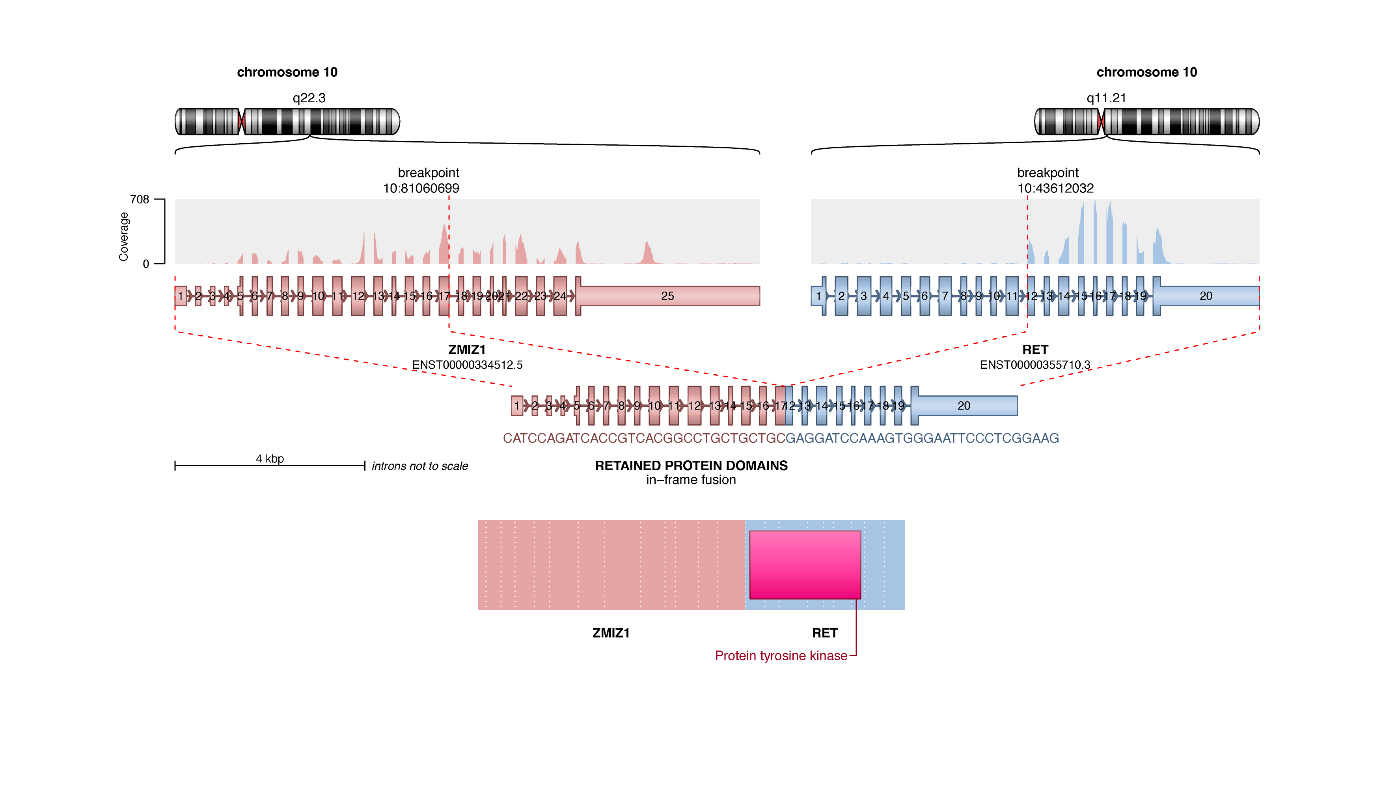


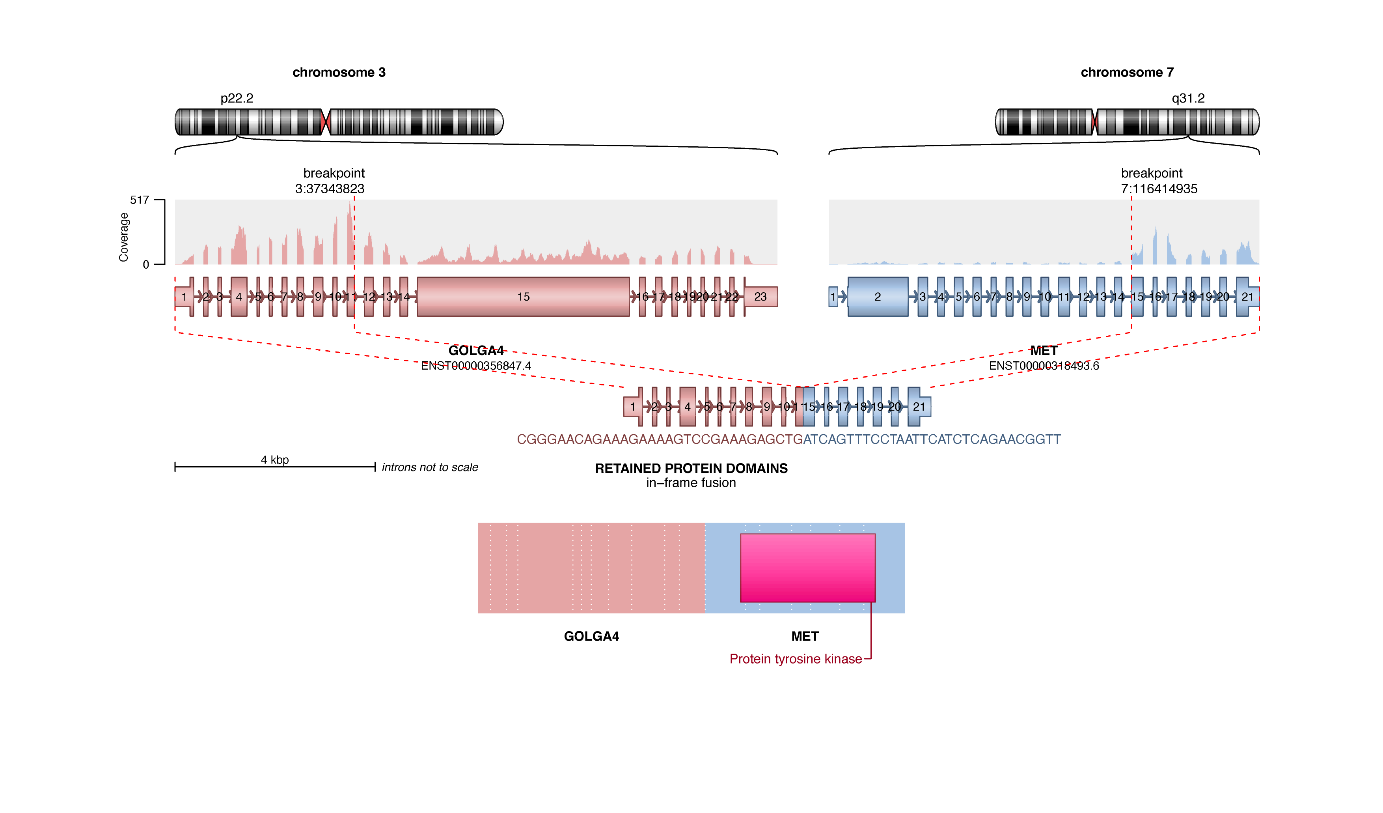


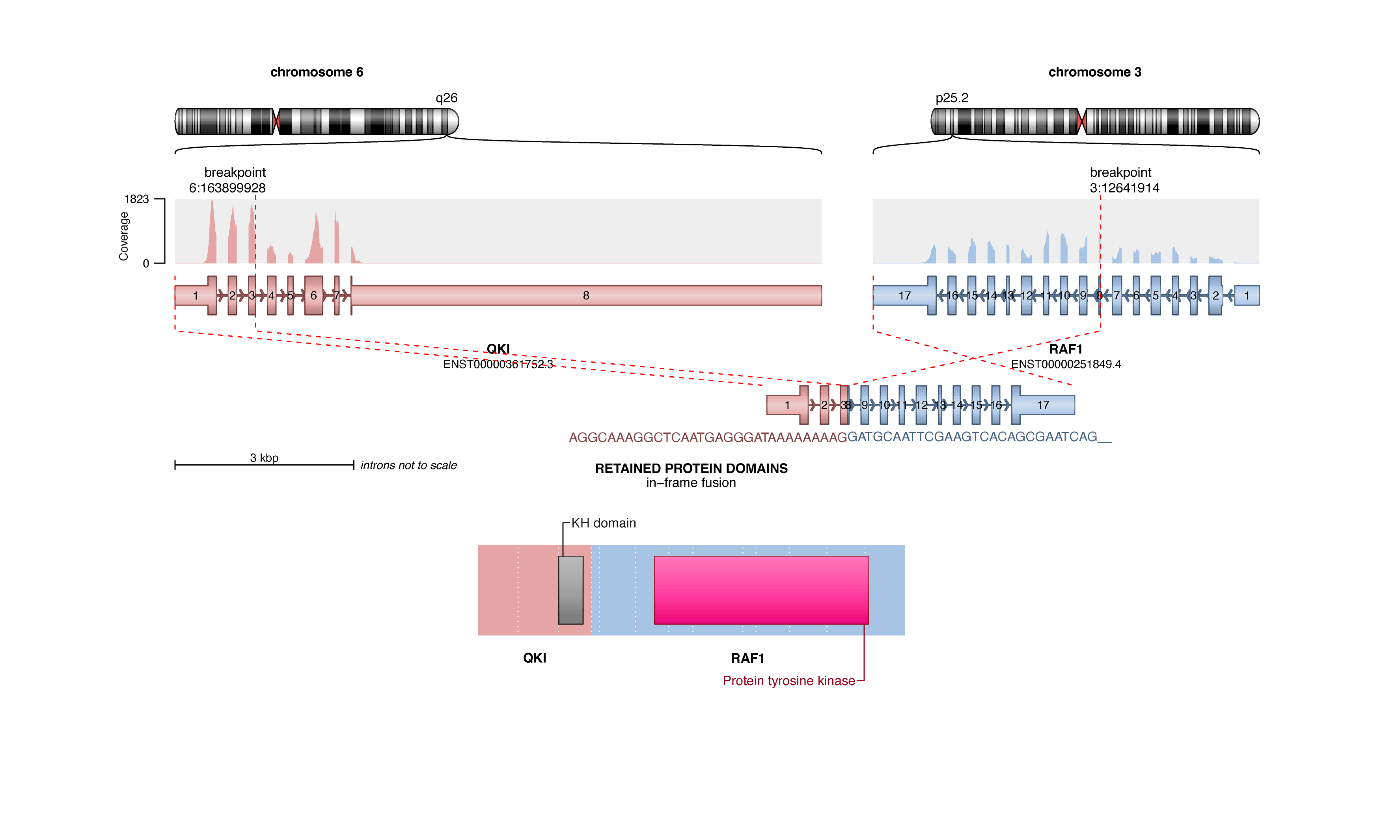

Supplement: Supplementary file 1 — Supplementary file1 (DOCX 1426 KB) [file 401_2022_2492_MOESM1_ESM.docx]
